# *In situ* sustained release of icariin from injectable thermosensitive PLGA-PEG-PLGA hydrogels combined with core decompression for steroid-induced osteonecrosis of femoral head

**DOI:** 10.3389/fphar.2025.1643316

**Published:** 2025-09-02

**Authors:** Yingxing Xu, Kaige Xu, Zehua Wang, Xinyu Tang, Yaping Jiang, Tao Li

**Affiliations:** ^1^ Department of Joint Surgery, The Affiliated Hospital of Qingdao University, Qingdao, Shandong, China; ^2^ Department of Orthopaedics, First Affiliated Hospital of Kunming Medical University, Kunming, Yunnan, China; ^3^ Department of Oral Implantology, The Affiliated Hospital of Qingdao University, Qingdao, Shandong, China

**Keywords:** icariin, PLGA-PEG-PLGA hydrogels, local sustained-release drug delivery system, osteonecrosis of femoral head, bone marrow mesenchymal stem cells

## Abstract

**Background:**

Icariin (ICA) has showed the beneficial effects on preventing the occurrence of steroid-induced osteonecrosis of the femoral head (SONFH) through enhancing bone formation and remodeling. In addition, the glucocorticoid-induced inhibition of cell proliferation and apoptosis are closely related to the pathogenesis of SONFH. In view of this, the present study was first designed to observe the effect of ICA on dexamethasone (Dex)-induced BMSCs and further reveal the relevant molecular mechanism. Furthermore, due to that the traditional oral administration of ICA is difficult to be absorbed and has a low bioavailability, the sustained-release ICA delivery system co-loaded thermosensitive PLGA-PEG-PLGA hydrogels was constructed, and the efficiency of this drug delivery system for the treatment of early SONFH was evaluated in rats model.

**Methods:**

The anti-apoptotic effect of ICA on the Dex-induced BMSCs was observed by crystal violet staining assay, Hoechst 33342 staining and flow cytometry analysis. Meanwhile, the protein expression levels of Akt/Bad/Bcl-2 signaling pathway were detected by Western blotting. Moreover, the sustained-release ICA delivery system co-loaded thermosensitive PLGA-PEG-PLGA hydrogels was constructed, and the sol-gel transition, *in vitro* degradation, as well as the sustained release of ICA from this drug delivery system was evaluated by a high performance liquid chromatography (HPLC) system. Ultimately, the sustained-release 2000 μM ICA delivery system co-loaded 25 wt% thermosensitive PLGA-PEG-PLGA hydrogels was injected into the femoral head and medullary cavity after core decompression (CD) to systematically assess the efficiency of this drug delivery system for the treatment of SONFH in rats model by MRI, Micro-CT and histological analysis.

**Results:**

ICA could rescue BMSCs from Dex-induced apoptosis through promoting the phosphorylation of Akt/Bad/Bcl-2 signaling pathway. Furthermore, the degradation of copolymer was related to the ICA concentration, and the sustained-release effect of this delivery system *in vitro* was influenced by the drug and gel concentration. Importantly, the local injection of the sustained-release ICA delivery system co-loaded thermosensitive PLGA-PEG-PLGA hydrogels combined with core decompression (CD) could significantly relieve the bone marrow edema, augment the trabeculae bone, reduce the empty lacunae, and decrease the accumulation of adipocyte while increasing the expression of Runx-2 and inhibiting the expression of PPAR-γ in the femoral head.

**Conclusion:**

Our data showed that local injection of this sustained-release drug delivery system combined with CD could significantly relieve the glucocorticoid-induced early osteonecrosis in the rats model with SONFH by increasing the residence time of ICA in the necrotic area of femoral head to maximize the anti-apoptotic, pro-osteogenic and anti-adipogenic effects of ICA.

## Introduction

As a progressive disorder characterized by subchondral necrosis, steroid-induced osteonecsis of the femoral head (SONFH) often presents with severe hip discomfort, dysfunction and even lameness (Mont and Hungerford, 1995). Total hip arthroplasty (THA) is the standard treatment for SONFH, but the results in young people are typically hampered by prosthesis-related problems (Radl et al., 2005; Hartley et al., 2000), despite the fact that it can provide significant pain relief and improve hip function in patients with SONFH. Therefore, more and more attention has been attracted in hip-preserving surgery for SONFH, such as core decompression (CD) (Mukisi-Mukaza et al., 2009; Rajagopal et al., 2012), bone grafting (Eisenschenk et al., 2001; Judet and Gilbert, 2001), osteotomy (Mont et al., 1996; Sakano et al., 2004) and stem cell therapy (Hernigou et al., 2015; Lau et al., 2014). Unfortunately, these approaches are often not satisfactory, which is attributed to the unclear pathogenesis of SONFH.

Icariin (ICA; C_33_H_40_O_15_; molecular weight: 676.67 g/mol), one of the major bioactive compounds extracted from epimedium-derived flavonoids, has been confirmed to play a significantly important role in the modulation of bone formation and remodeling *in vivo* or *in vitro* (Wang et al., 2018; Nian et al., 2009; Poon et al., 2024; Zhang et al., 2024; Liu et al., 2024), which is of great significance not only for the treatment of osteoporosis, but also for that of SONFH. Increasing number of studies have showed the beneficial effects of ICA on preventing the occurrence of SONFH in rats model, such as inhibiting bone loss (Huang et al., 2018), reducing the level of thrombomodulin (TM) and vascular endothelial growth factor (VEGF) in serum (Yue et al., 2021), improving the blood vessel volume and decreasing the empty lacunae formation of femoral head (Yu et al., 2019). It should be noted that ICA was administered orally in these studies *in vivo*. However, one study for the metabolism of ICA (42 mg/g) showed that the plasma and tissue concentration of ICA in rat were maintained at a lower level until 24 h after oral administration, suggesting oral administration of ICA was difficult to be absorbed and had a low bioavailability (Xu et al., 2017). As a result of this, several studies have tried to verify the efficacy of ICA after local administration. Zhang et al. (2018) reported that in the treatment of periodontitis, local injection of ICA improved the regeneration of periodontal tissue and the regression of inflammation. Furthermore, Wu et al. (2017) constructed the calcium phosphate cement scaffolds loaded with ICA, and then implanted it into the calvarial defect of rats model, and found that this local application of ICA could promote the osteogenesis and angiogenesis of local region to repair bone defects. Similarly, the study of Zhao et al. (2010) also confirmed the local osteoinductive effect of ICA-CPC tablets in a mouse calvarial defect model. Thus, the local administration of ICA has exhibited several promising prospects in tissue engineering of bone as a osteoinductive compound.

Poly-(_D,L_-lactic acid-co-glycolic acid) (PLGA)-polyethylene glycol (PEG)-PLGA (PLGA-PEG-PLGA) hydrogel, a triblock copolymer with hydrophobic and hydrophilic core, has showed an excellent performance for thermosensitive controlled-release of drug, as well as the good biodegradability, biocompatibility, conveniency and low toxicity (Chan et al., 2019; Ma et al., 2014; Wang et al., 2017), and thus has been widely used in injectable drug delivery systems (Wang et al., 2017). In addition, the components of PLGA-PEG-PLGA could be tailored to provide various phase transition temperature in order to meet the practical requirements (Gao et al., 2011; Gao et al., 2010).

Our previous study has confirmed the roles of ICA on promoting the osteogenesis and inhibiting the adipogenesis of bone marrow mesenchymal stem cells (BMSCs) through activation of the miR-23a/Wnt/β-catenin signaling pathway axis (Xu et al., 2021a). In addition to causing an imbalance in the osteogenic and lipogenic differentiation of BMSCs, glucocorticoid-induced inhibition of cell proliferation and apoptosis are also implicated in the pathogenesis of SONFH (Xu et al., 2020a). In view of this, the present study was designed to observe whether ICA could rescue BMSCs from dexamethasone (Dex)-induced apoptosis and further reveal the relevant molecular mechanism. Importantly, the sustained-release ICA delivery system co-loaded thermosensitive PLGA-PEG-PLGA hydrogels was constructed and then injected into the femoral head and medullary cavity after core decompression (CD) to systematically assess the efficiency of this drug delivery system for the treatment of early SONFH in rats model.

## Materials and methods

### Isolation and culture of BMSCs

According to our previous report, BMSCs were obtained by first isolating bone marrow cells from the bilateral femur and tibia of Sprague Dawley (SD) rats, followed by purification using a cell apposition method (Xu et al., 2012). In brief, SD rats were euthanized by carbon dioxide asphyxiation, and then were completely immersed in 75% ethanol solution. After sterilization for 10 min, the bilateral femurs and tibias were obtained and rinsed three times in sterile PBS to remove residual ethanol. The medullary cavity was exposed after the epiphysis at both ends were cut off, and then was repeatedly washed with the complete medium containing low glucose DMEM (Solarbio, Beijing, China), 10% fetal bovine serum (FBS) (Gibco, Australia) and 100 units/ml penicillin and streptomycin (Solarbio, Beijing, China) by a 5 mL syringe. After that, the flushing solution was collected and centrifuged at 1,000 rpm for 5 min to collect cells. After washing with PBS for 3 times, cells were seeded in 25 cm^2^ cell culture bottle at a higher density and incubated with 5% CO_2_ at 37 °C. The culture medium was changed for the first time after 3 days, and then once every other day. When the confluence reached more than 90%, cells were digested with 0.25% trypsin containing EDTA and collected, and then were subcultured in 1:2 ratio. The third passage cells were utilized in subsequent experiments.

### Phenotyping of BMSCs

The surface markers expression of the third passage cells was investigated by Apogee A50-MICRO flow cytometer (Apogee, United Kingdom) according to the following procedure. Briefly, after the cells were collected and rinsed with pre-cold PBS three times, the cell suspension was incubated with CD34-Phycoerythrin (PE), CD45-PE, CD73-fluorescein isothiocyanate (FITC), CD90-FITC for 30 min in dark at 37 °C respectively. Untreated cells were used as control. All antibodies were provided by BD Biosciences (Franklin Lakes, United States).

### Osteogenic and adipogenic differentiation of BMSCs

The potential of the third passage cells in osteogenic and adipogenic differentiation were evaluated using differentiation medium (Fuyuanbio, Shanghai, China) as the manufacturer’s instructions described. Osteogenic induction experiment was performed, where cells were treated with osteogenic differentiation medium when they reached 60% confluence, and then stained with alkaline phosphatase (Solarbio, Beijing, China) 14 days after induction. Adipogenic induction experiment was performed, where cells were treated with adipogenic differentiation medium when they reached 90% confluence, and then stained with oil red O (Solarbio, Beijing, China) 21 days after induction.

### Treatments of BMSCs

To investigate the anti-apoptotic effect and related molecular mechanism of ICA on Dex-induced BMSCs, cells were divided into the following seven groups. (1) Dex group: cells were treated with 10^–6^ mol/L Dex; (2) Dex +10 μM ICA group: cells were treated with 10^–6^ mol/L Dex and 10 μM ICA; (3) Dex +20 μM ICA group: cells were treated with 10^–6^ mol/L Dex and 20 μM ICA; (4) Dex +40 μM ICA group: cells were treated with 10^–6^ mol/L Dex and 40 μM ICA; (5) Dex +20 μM ICA + MK-2206 group: cells were treated with 10^–6^ mol/L Dex, 20 μM ICA and 5 μmol/L MK-2206 (cell signaling, United States) used as a selective inhibitor of Akt; (6) Dex + SC79 group: cells were treated with 10^–6^ mol/L Dex and 8 μg/mL SC79 (cell signaling, United States) used as a unique specific activator of Akt; (7) Control group: cells were cultured in complete medium.

### Crystal violet staining assay for BMSCs proliferation

Crystal violet staining assay was used to observe the proliferation of BMSCs from day 1–7. BMSCs were seeded into 96-well plates at a density of 5 × 10^3^ cells/well, and then were treated continuously for 7 days as described in the above groups. During this time, crystal violet staining assay was performed every day. In detail, cells were fixed with 4% paraformaldehyde for 10 min, and stained with 0.25% crystal violet solution for 30 min. After washing with PBS for 3 times, the stained cells were observed under an inverted phase-contrast microscope. Then, 200 μL of methanol was added to each well and the absorbance of each well was measured at 570 nm using a microplate reader (Tecan, Austria) after fully dissolving the crystal violet.

### Morphological assessment of apoptotic BMSCs

The chromatin dye Hoechst 33342 kit (Solarbio, Beijing, China) was used to assess the morphology of apoptotic cells. In brief, BMSCs were seeded into 24-well plates at a density of 5 × 10^4^ cells/well, and then were stained with chromatin dye Hoechst 33342 at 4 °C for 20 min following continuous treatment for 7 days as described in the above groups. After that, such the morphological characteristics of apoptotic cells as karyopyknosis, chromatic agglutination and nuclear fragmentation, were identified by a fluorescent microscopy.

### Flow cytometry analysis for BMSCs apoptosis

To evaluate the percentage of apoptotic cells, the flow cytometry analysis was performed by Annexin V-PE/7-AAD kit (BD Biosciences, United States). In brief, BMSCs were seeded into 6-well plates at a density of 2 × 10^5^ cells/well. After continuous treatment for 7 days as described in the above groups, cells were collected and washed with binding buffer for 3 times. Then, cells were stained with Annexin V-PE and 7-AAD respectively at 5 µL/10^5^ cells/100 µL in a dark at room temperature. After that, the stained cells were analyzed and counted by Apogee A50-MICRO flow cytometer (Apogee, United Kingdom).

### Western blotting analysis

Western blotting analysis was used to detect the protein expression level of Akt/Bad/Bcl-2 signal pathway, including Akt, phosphorylated Akt (p-Akt), Bad, phosphorylated Bad, Bcl-2, caspase-3 and cleaved-caspase-3. In brief, BMSCs were seeded into 6-well plates at a density of 2 × 10^5^ cells/well. After continuous treatment for 3 days as described in the above groups, the total protein of BMSCs in each group were extracted using the pre-cold RIPA Lysis Buffer (Solarbio, Beijing, China) containing 1% protease inhibitor cocktail (MCE, Shanghai, China). The cell lysates were collected and heated at 95 °C for 5 min after mixing with protein loading buffer (EpiZyme, Shanghai, China). Protein samples from each group were equivalently separated by SDS-PAGE (EpiZyme, Shanghai, China) and the separated protein samples were then electrotransferred to PVDF membranes (Merck-Millipore, France). Subsequently, PVDF membranes were blocked by 5% fat-free milk in TBST (Solarbio, Beijing, China) for 2 h at 37 °C and then incubated with primary antibody overnight at 4 °C. The following day, PVDF membranes were soaked in secondary antibody solution (HRP-conjugated) for 1 h at 37 °C after completely washing off the primary antibody. Target bands were scanned by BioSpectrum imaging system (UVP, United States) followed by visualisation using ECL-PLUS reagent (Merck-Millipore, France). The scans were finally quantified by integrated density obtained from ImageJ software (vesion 1.52u) and then normalised by GAPDH.

Except the primary antibody for GAPDH (Elabscience, China) and all second antibodies (Elabscience, China), the other primary antibodies, such as anti-Akt and p-Akt, anti-Bad and p-Bad, anti-Bcl-2, anti-caspase-3 and cleaved-caspase-3 antibody were purchased from Cell Signaling Technology (Danvers, United States). Antibody dilution buffer (Boster Biological Technology, Inc.) was used to dilute all primary and second antibodies at a ratio recommended by the manufacturer’s instructions.

### Synthesis of the copolymers

The PLGA-PEG-PLGA triblock copolymer [lactic acid (LA):glycolic acid (GA) = 5:1, PEG:PLGA = 3:7] was provided by Daigang biological company (Jinan, China) and was synthesized by the ring-opening polymerization as Zentner et al. (2001) described previously. Briefly, each component was added into the polymerization reaction bottle in corresponding proportion, and then stannous octoate was used as the catalyst. After repeatedly passing nitrogen and vacuumizing to remove trace water and oxygen, the tube is sealed and placed in the set temperature to obtain the PLGA-PEG-PLGA triblock copolymer. Following dissolving in water, the polymer was purified by phase separation at elevated temperature and dried in vacuum to constant weight.

### Preparation of the sustained-release ICA delivery system co-loaded copolymer solution

According to the previous study (Wu et al., 2017), three concentrations of ICA (200, 2,000 and 20,000 μM, respectively) were selected to be loaded in the PLGA-PEG-PLGA triblock copolymer based on the previous method (Gao et al., 2010). In detail, ICA dissolved in DMSO (Solarbio, Beijing, China) was added to 25 wt% PLGA-PEG-PLGA copolymer aqueous solution (the copolymer was dissolved in PBS in the ratio of 1:5 at 4 °C, and was filtered with a 0.22 μm membrane) and homogenized at 8,000 rpm for 40 s until the solution was homogeneous and clear at room temperature.

In addition, ICA-loaded copolymer solutions (2000 μM) with copolymer concentrations of 15, 20 and 25 wt% were prepared respectively, due to the copolymer with a concentration of less than 25 wt% could be dissolved thoroughly.

### *In vitro* copolymer degradation

Four concentrations of ICA (0, 200, 2000 and 20000 μM) loaded in the 25 wt% PLGA-PEG-PLGA triblock copolymers (0.5 mL) were transferred into four sealed vials respectively using 1 mL syringe, and then were incubated at 37 °C until hydrogels were formed. After that, 3 mL of simulated body fluid (FBS) was added into the vials to cover hydrogels completely, and the vials were placed at 37 °C and shook at 50 rpm continuously. At predetermined time points (1, 3, 5, 7, 14, 21, 28, 35 and 42 days), the undegraded copolymers were accurately weighed after all the buffer was removed thoroughly.

### *In vitro* release

Three concentrations of ICA (200, 2000 and 20000 μM) loaded in the 25 wt% PLGA-PEG-PLGA triblock copolymers (2 mL) and ICA-loaded copolymer solutions (2000 μM) with copolymer concentrations of 15, 20 and 25 wt% (2 mL) were transferred into the cell culture dishes respectively, and then were incubated at 37 °C until hydrogels were formed. After that, 5 mL of simulated body fluid (FBS) was added into the dishes to cover hydrogels completely, and the dishes were placed at 37 °C and shook at 50 rpm continuously. At each predetermined time points (1, 3, 6, 9, 12, 24 h and 3, 5, 7, 14, 21, 28 days), the 4 mL of supernatant was collected after full resuspension, and then the equivalent fresh SBF was added to the dishes to cover hydrogels completely. The released ICA was quantified by an a high performance liquid chromatography (HPLC) system (Thermofisher U3000, United States), and the determination conditions were as follows: chromatographic column was An ZORBAX SB-C18 column (250 mm × 4.6 mm, 5 μm); column temperature was maintained at 25 °C; the flow rate was 1 mL/min; mobile phase A was acetonitrile and mobile phase B was water (the ratio was 26:74); the detection wavelength was 270 nm; the injection volume was 10 μL. Then, the data were conducted by the following formula to calculate the cumulative amount of release for ICA:

$$\text{Cumulative amount of release }\left(\%\right)=100\times M_{t}/M_{\infty }$$

Where M_t_ was the amount of ICA released from copolymer at each predetermined time points, and $M_{\infty }$ was the total amount of ICA added in copolymer.

### Animals grouping and treatment

**FIGURE 1:**
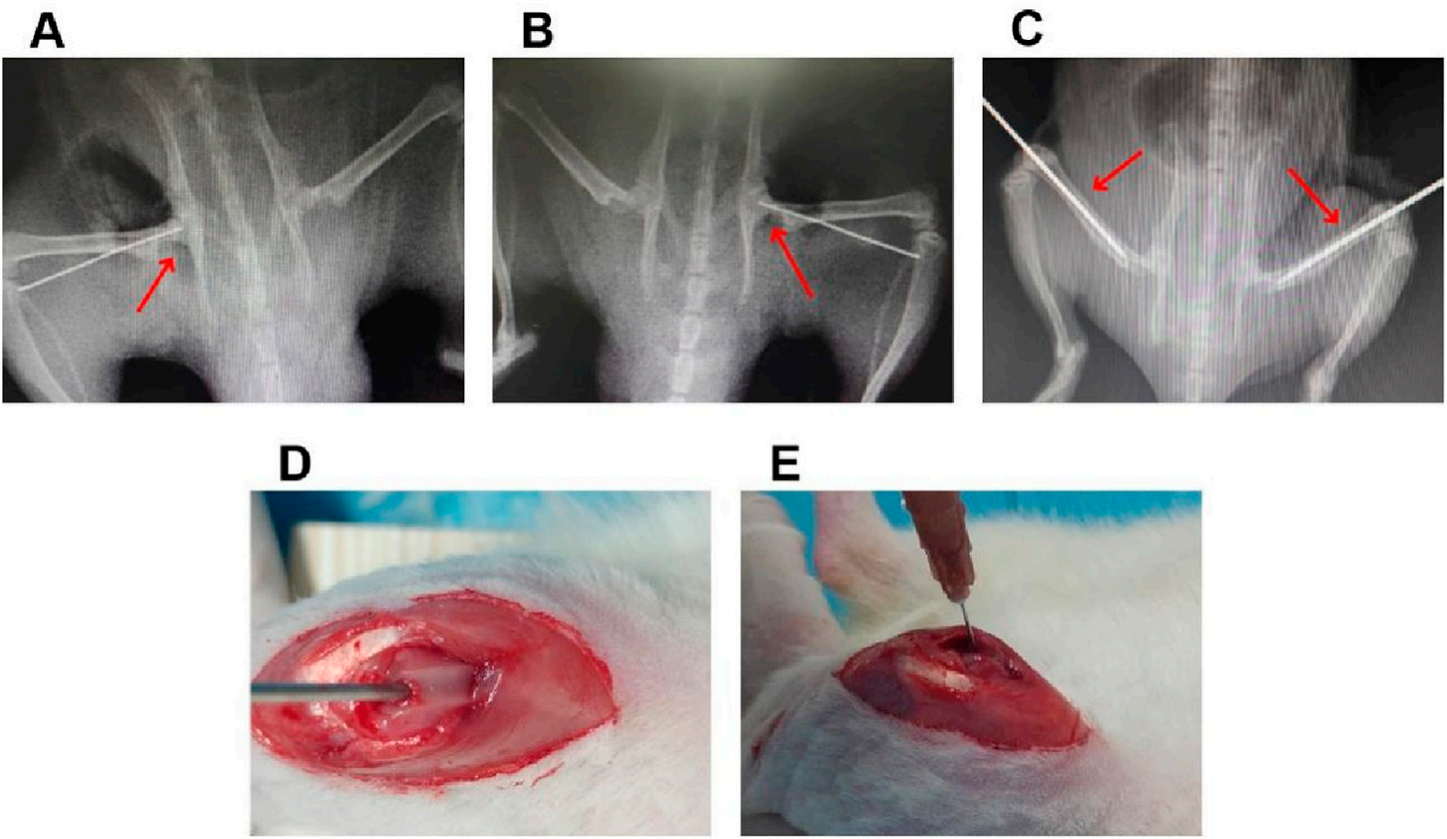
The details of local injection of the sustained-release ICA delivery system co-loaded thermosensitive PLGA-PEG-PLGA hydrogels combined with CD for the treatment of in the rats model with SONFH. **(A,B)** The representative radiological images for the CD operation. **(C)** The representative radiological images for the intramedullary puncture. **(D,E)** Representative operation images for the intramedullary injection.

**FIGURE 2:**
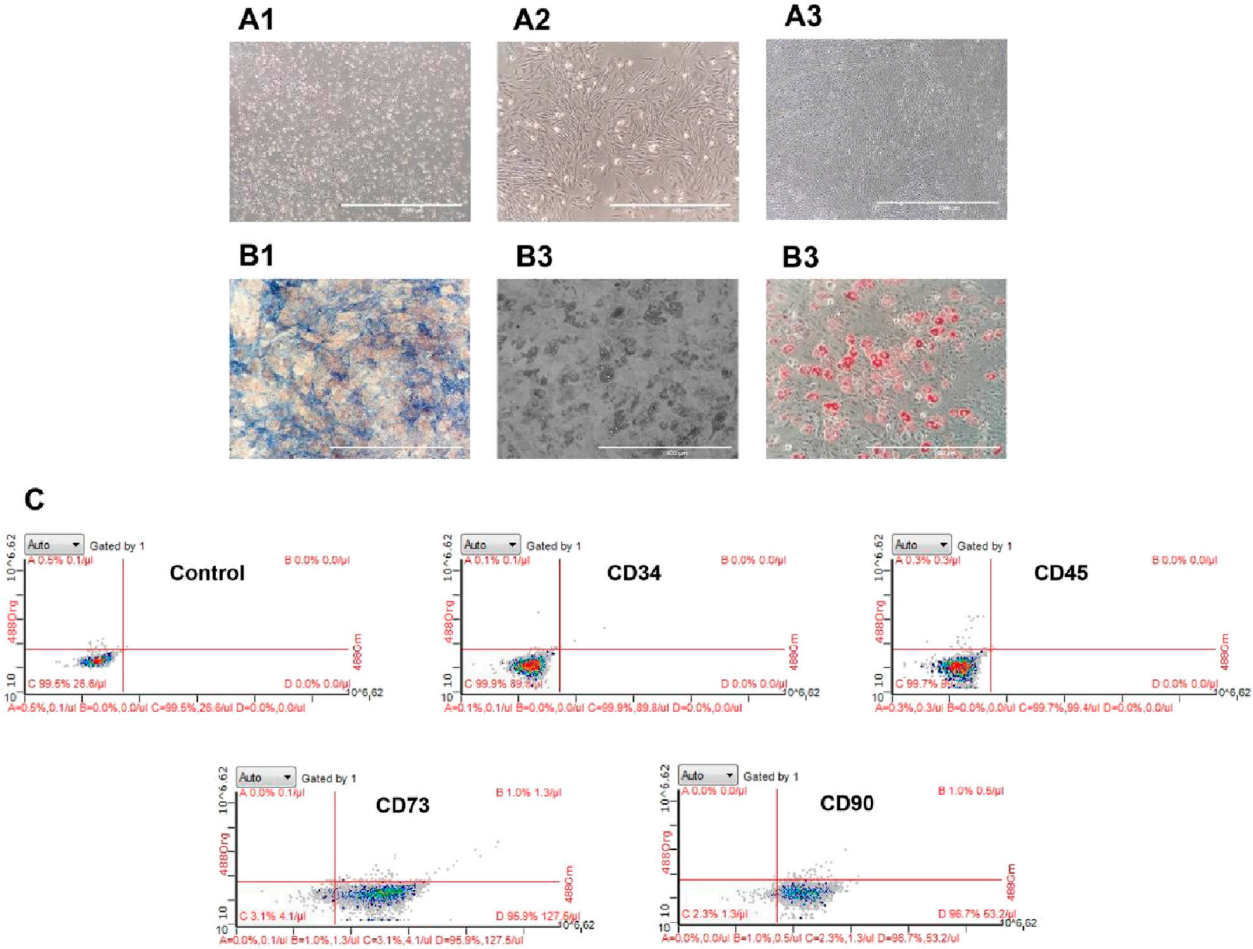
Identification of BMSCs. **(A)** Representative morphology of BMSCs derived from rats under an inverted phase contrast microscope (scale bar = 1000 um): **(A1)** the first passage cells; **(A2)** the cecond passage cells with confluency of 80%; **(A3)** the thirs passage cells with confluency of 100%. B. Identification of BMSCs for osteogenesis and adipogenesis: **(B1)** ALP staining (scale bar = 1000 um); **(B2)** lipid droplets formation (scale bar = 200 um); **(B3)** oil red O staining (scale bar = 200 um). **(C)** Flow cytometry analysis for the surface markers of BMSCs (CD34, CD45, CD73 and CD90).

A total of 50 healthy male SD rats of 300–350 g at 8 weeks of age were used to investigate the efficiency of the sustained-release ICA delivery system co-loaded thermosensitive PLGA-PEG-PLGA for the treatment of SONFH in rats model. Among of them, 40 rats were given a gluteal injection of 20 mg/kg/day of methylprednisolone (Pfizer, New York, United States) for 3 days/week for 3 weeks, and the others were treated with equivalent normal saline as a control. After treatment for 6 weeks, these rats were anesthetized, and then examined by magnetic resonance imaging (MRI) to confirm the validity of SONFH model. After that, all rats in the present study were divided into the following five groups randomly. (1) 2000 μM ICA + PLGA + CD + ONFH group: rats with SONFH were treated with the core decompression (CD) of femoral head combined with injection of sustained-release ICA delivery system co-loaded thermosensitive PLGA-PEG-PLGA (n = 10). In detail, after the rats with SONFH were anesthetized with 0.3% pentobarbital sodium (1 mL/100 g), the core decompression of femoral head was performed with the assistance of fluoroscopy, and then 2000 μM ICA co-loaded PLGA-PEG-PLGA copolymer (0.2 mL) were injected into femoral medullary cavity through the minimally invasive incision of distal femur (Figure 1); (2) 2000 μM ICA + CD + SONFH group: rats with SONFH were treated with the core decompression of femoral head combined with injection of 2000 μM ICA solution (0.2 mL). The detailed operation was as described above (n = 10); (3) PLGA + CD + SONFH group: rats with SONFH were treated with the core decompression of femoral head combined with injection of PLGA-PEG-PLGA copolymer (0.2 mL). The detailed operation was as described above (n = 10); (4) CD + SONFH group: rats with SONFH were treated with the core decompression of femoral head combined with injection of normal saline (0.2 mL). The detailed operation was as described above (n = 10); (5) Control group: untreated healthy rats served as controls (n = 10). After 12 weeks of the above treatment, the femoral heads were obtained from the rats executed by CO2 asphyxiation for radiographic examination (MRI and micro-CT) and pathological examination (HE staining and immunohistochemistry).

All rats in this study were purchased from Pengyue Laboratory Animal Breeding Ltd. (Jinan, China). All subsequent animal experiments followed the Guide for the Care and Use of Laboratory Animals and were carried out in an animal laboratory with a Specific Pathogen Free (SPF) class.

### MRI analysis

The Skyra 3.0T MRI system with a phased-array body coil (Siemens, Germany) was used to investigate abnormal signals in each group of femoral heads. According to the parameters reported in our previous study (Xu et al., 2021b), the turbo spin-echo T2-weighted fat-saturated images in the transverse plane were obtained. The specific details of parameters were as follows: repetition time of 3,000 ms, echo time of 38 ms, slice thickness of 1.4 mm, interslice distance of 0.14 mm, view field of 120 mm, and matrix of 384 × 320 pixels.

### Micro-CT analysis

After soft tissue removal, the femoral heads were analyzed with μCT100 (SCANCO MEDICAL, Zurich, Switzerland) to visualize the local lesions of the femoral head after fixation with 4% paraformaldehyde. The micro-CT scan parameters were set according to our previous report (Xu et al., 2021b), and the details of parameters were as follows: scan energy intensity of 70 KVp and 200 μA, filter of 0.5 AL, matrix of 1,022 mm × 1,022 mm, view field of 15.1 mm, resolution of 14.8 μm, and sampling time of 250 ms. Subsequently, two-dimensional images of the femoral head and analytical data of the femoral bone trabeculae, such as Tb.Th, Tb. Sp, BV/TV, Tb.N and bone mineral density (BMD), were acquired by the micro-CT system and SCANCO μCT Assessment Program V6.6 software respectively.

### Histological analysis

The collected femoral heads were removed from the soft tissue and fixed in 4% paraformaldehyde. Subsequently, the specimens were decalcified with 10% EDTA for 4 weeks, and then were dehydrated and embedded in paraffin. The coronal paraffin sections of 5 µm were then made using a sectioning machine (Leica, Biocut, Germany), and then were performed by hematoxylin and eosin (HE) staining and IHC analysis for the expression of Runx2 and PPAR-γ after dewaxing. All photomicrographs were acquired using a panorama scanner (3DHISTECH P250 FLASH, Hungary).

### Statistical analysis

Three replicate experiments were carried out after three samples were analyzed in each group. The SPSS 19.0 software (IBM, United States) was used to perform statistical analysis of all data in this study. Data from more than two groups were analysed using one-way analysis of variance (ANOVA), with values expressed as mean ± standard deviation (SD). Tamhane’s T2 test was used when heteroscedasticity was found by conducting homogeneity of variance tests. A *p*-value of <0.05 was used as the statistical difference. GraphPad Prism 8 software (GraphPad, CA, United States) was used to produce the statistical graphs.

## Results

### Identification for BMSCs

The manifestations characterized by homogeneous fibroblast-like, spindle-shaped morphology were observed in the third passage cells (Figure 2A). Furthermore, ALP and oil red O staining have confirmed their potentials of osteogenic and adipogenic differentiation (Figure 2B). The flow cytometry analysis further showed that the isolated cells expressed typical surface markers for marrow derived stem cells with positivity for CD73 (95.9%), CD90 (96.7%), and negativity for CD34 (0.1%) and CD45 (0.3%), which were two specific cell surface markers of hematopoietic cells (Figure 2C).

### ICA enhanced the survival of Dex-induced BMSCs

**FIGURE 3:**
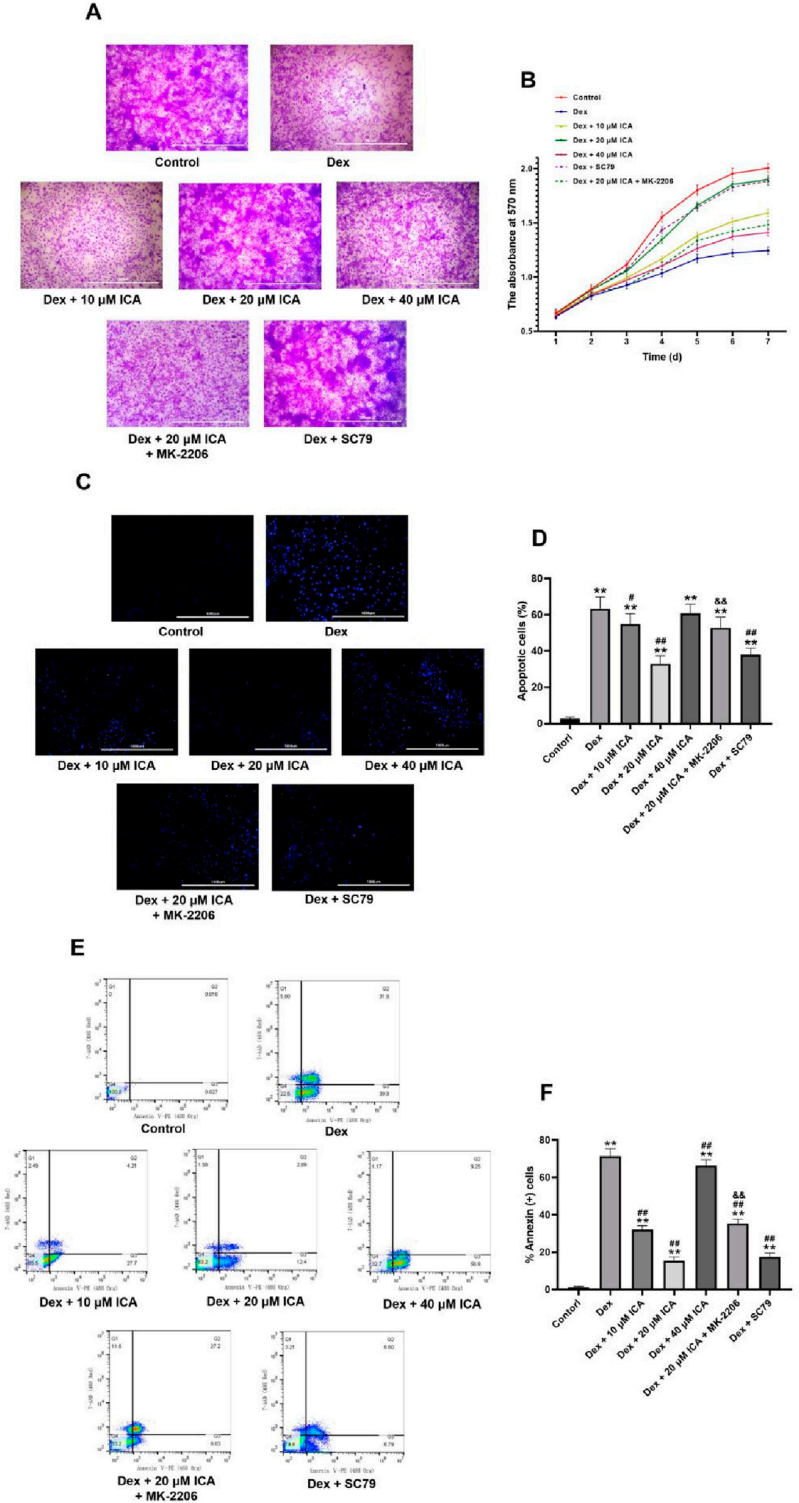
ICA enhanced the survival of Dex-induced BMSCs. **(A)** Representative images for crystal violet assay (scale bar = 200 um). **(B)** Absorbance curves for quantification of crystal violet assay. **(C)** Representative fluorescence microscope images for Hoechst 33342 staining (scale bar = 1,000 um). **(D)** Histogram for the percentage of apoptotic cells. **(E)** Representative images for flow cytometric analysis: Q1-Q4 showing the percentage of necrotic cells, late apoptotic cells, early apoptotic cell and normal cells, respectively. **(F)** The total percentage of apoptotic cells in Q2 and Q3. Note: all data were expressed as mean ± SD after three replicates. ^**^ *P* < 0.01 compared with the control group, ^#^ *P* < 0.05 compared with the Dex group, ^##^ *P* < 0.01 compared with the Dex group. ^&&^ *P* < 0.01 compared with the Dex +20 μM ICA group.

The proliferation of Dex-induced BMSCs were investigated by crystal violet staining assay. As shown in Figures 3A,B, 10^–6^ mol/L Dex inhibited the proliferation of BMSCs remarkablely in a time-dependent manner from 1 to 7 days, which was consistent with our previous studies (Xu et al., 2020a). Significantly, ICA antagonized the inhibitory effect of Dex on the BMSCs proliferation over the range of 10–40 μM. It was noteworthy that this effect of ICA was not dose-dependent, but declined at the concentration of 40 μM.

Furthermore, the apoptosis of Dex-induced BMSCs were assessed by Hoechst 33342 staining and flow cytometry analysis. As shown in Figures 3C–F, 10^−6^ mol/L Dex induced the apoptosis of BMSCs in a time-dependent manner from 1 to 7 days in accordance with our previous studies (Xu et al., 2020a). Similarly, ICA rescued the BMSCs from Dex-induced apoptosis at the concentration of 10 and 20 μM, rather than at the concentration of 40 μM.

### ICA promoted the phosphorylation of Akt/Bad/Bcl-2 signaling pathway in the Dex-induced BMSCs

**FIGURE 4:**
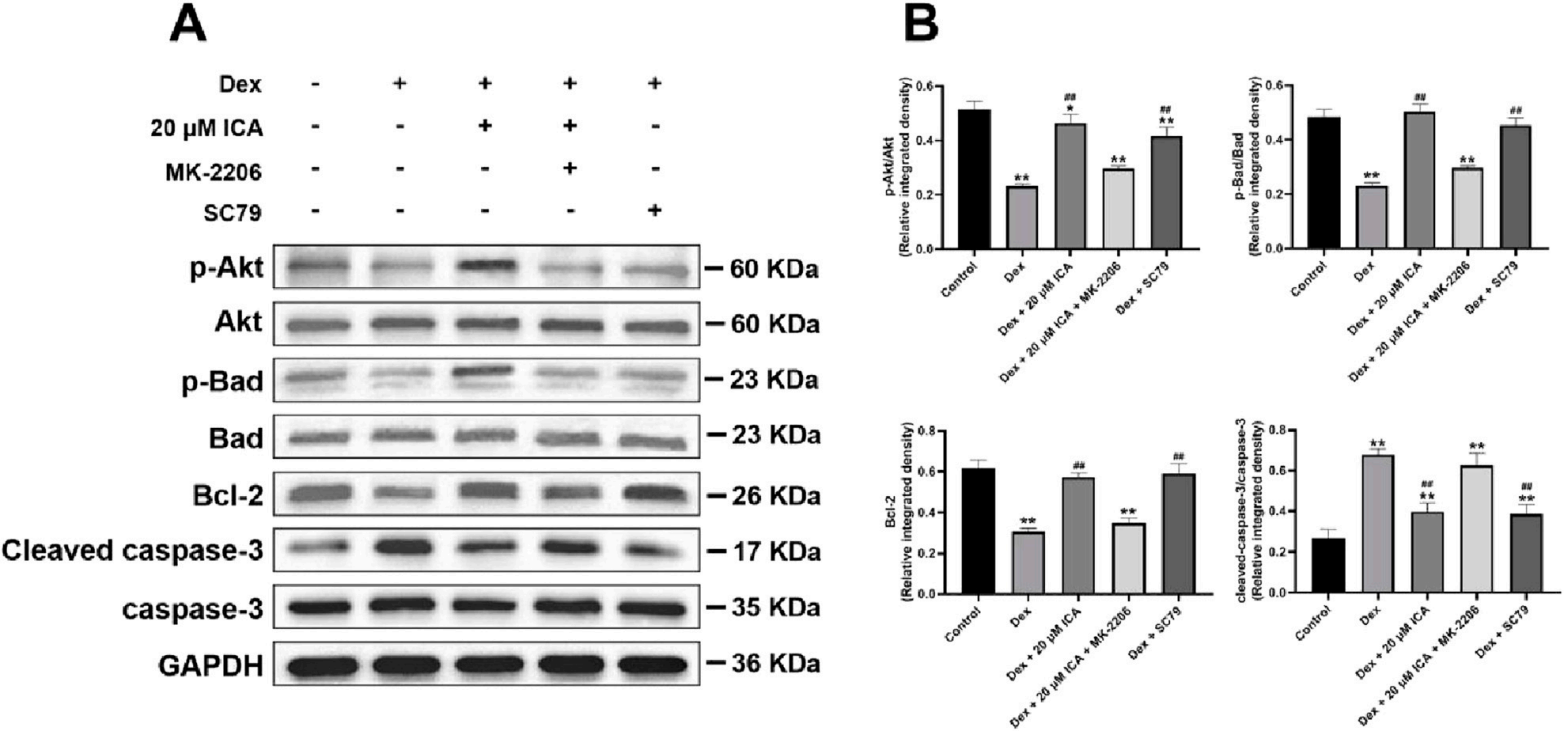
ICA promoted the phosphorylation of Akt/Bad/Bcl-2 signaling pathway in the Dex-induced BMSCs. **(A)** Representative images for Western blotting. **(B)** Histogram for quantification of integrated density in target bands normalized to GAPDH. Note: all data were expressed as mean ± SD after three replicates. ^*^ *P* < 0.05 compared with the control group, ^**^ *P* < 0.01 compared with the control group, ^##^ *P* < 0.01 compared with the Dex group.

To further reveal the anti-apoptotic mechanism of ICA on Dex-induced BMSCs, SC79 and MK-2206 served as the unique specific activator and selective inhibitor in the antagonism and activation experiment for Akt/Bad/Bcl-2 signaling pathway, respectively. As shown in Figure 3, ICA had a similar effect to SC79 against Dex-induced apoptosis in BMSCs, while this effect could be blocked by MK-2206. Moreover, Western blotting analysis suggested that 20 μM ICA could promote the phosphorylation of Akt and Bad, enhance the expression of Bcl-2, and suppress the level of cleaved casapase-3 in Dex-induced BMSCs. Importantly, this effect of ICA on the Akt, Bad, Bcl-2 and casapase-3 also resembled the that of SC79, but could be suppressed by MK-2206. Taken together, ICA promoted the phosphorylation of Akt/Bad/Bcl-2 signaling pathway to prevent Dex-induced BMSCs from apoptosis (Figure 4).

### Sol-gel transition and *in vitro* degradation of PLGA-PEG-PLGA hydrogels

**FIGURE 5:**
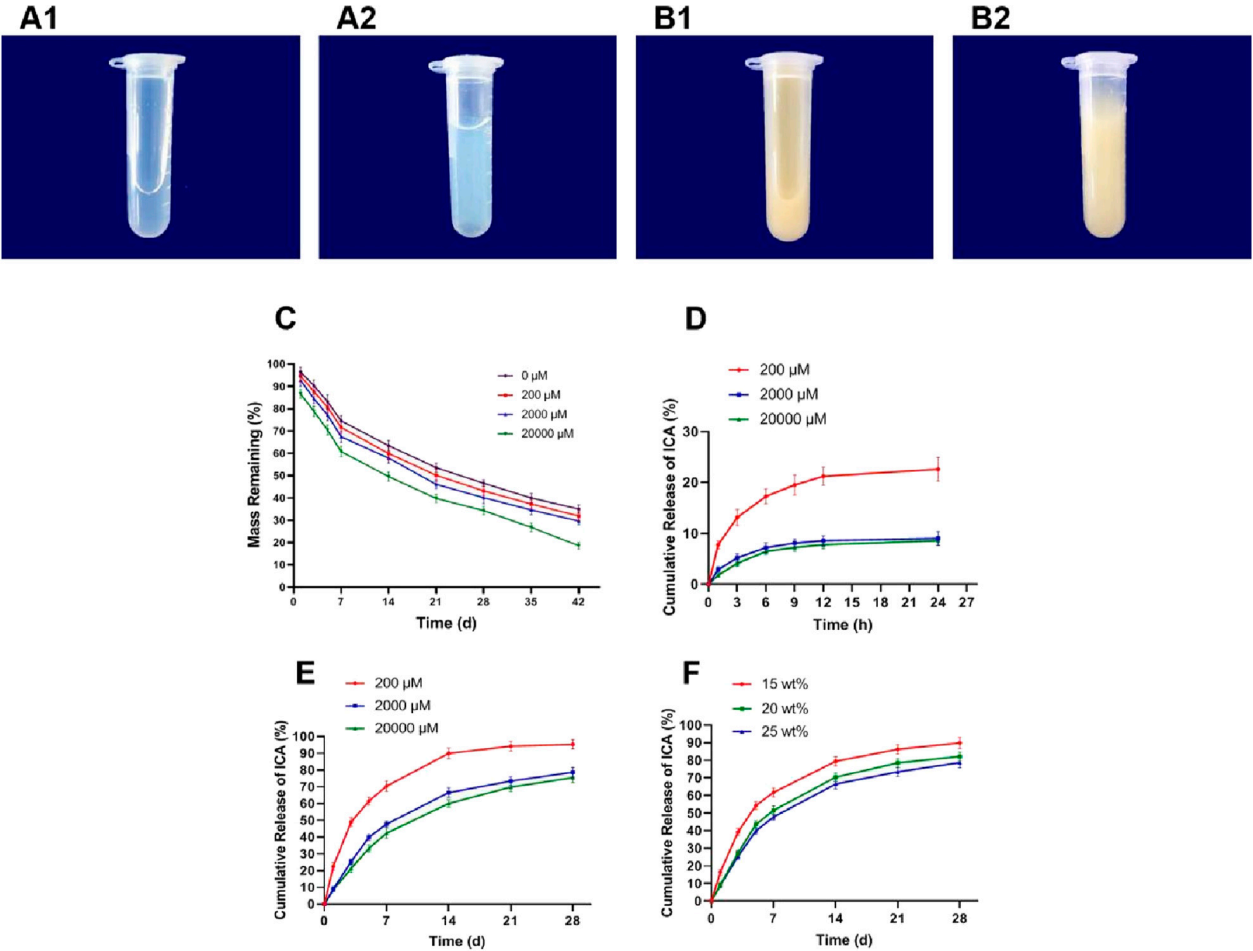
The characteristics of sustained-release ICA delivery system co-loaded thermosensitive PLGA-PEG-PLGA hydrogels. **(A,B)** Representative images showing the Sol-gel transition. A1. Status of thermosensitive PLGA-PEG-PLGA hydrogels at 4 °C. A2. Status of thermosensitive PLGA hydrogels at 37 °C. B1. Status of 20 μM ICA co-loaded thermosensitive PLGA-PEG-PLGA hydrogels at 4 °C. B2. Status of 20 μM ICA co-loaded thermosensitive PLGA-PEG-PLGA hydrogels at 37 °C. **(C)** The *in vitro* degradation of 25 wt% PLGA-PEG-PLGA hydrogels loaded by ICA at various concentrations. **(D)** The *in vitro* release profiles of various concentrations of ICA from 25 wt% PLGA-PEG-PLGA hydrogels within 24 h. **(E)** The *in vitro* release profiles of various concentrations of ICA from 25 wt% PLGA-PEG-PLGA hydrogels within 4 weeks. **(F)** The *in vitro* release profiles of 20 μM ICA from various concentrations of PLGA-PEG-PLGA hydrogels within 4 weeks.

Thermosensitive PLGA-PEG-PLGA hydrogels was used in this study for the convenience of injection *in vivo*. As shown in Figures 5A,B, the 25 wt% copolymer was a transparent solution at 4 °C, and could form homogenous translucent colloid at 37 °C rapidly. Likewise, 20 μM ICA-loaded 25 wt% copolymer also exhibited the sol-to-gel transition behavior in response to temperature.

Furthermore, we investigated the degradation of 25 wt% PLGA-PEG-PLGA hydrogels loaded by ICA at the concentration of 0, 200, 2000 and 20000 μM *in vitro.* As shown in Figure 5C, approximately 60% of the hydrogels without ICA loading were degraded at 42nd day. Significantly, the hydrogels degraded more faster with the increase of ICA concentration, and approximately 70% of the hydrogels loaded by 200 or 2000 μM ICA were degraded at the 42nd day, but more than 80% of the hydrogels were degraded loading with 20000 μM ICA.

### The sustained release of ICA from PLGA-PEG-PLGA hydrogels

To obtain the optimal proportion of the copolymer and the drug in the sustained-release ICA delivery system co-loaded PLGA-PEG-PLGA hydrogels, we observed the effect of ICA loading levels and copolymer concentration on the release patterns in a time-independence. Our previous study has confirmed that the optimal dosage of ICA on promoting the osteogenesis and inhibiting the adipogenesis of BMSCs *in vitro* was 20 μM (Xu et al., 2021a), therefore, the ICA at the concentration of 200, 2,000, 20,000 μM was used to construct the sustained-release drug delivery system respectively according to the previous report (Wu et al., 2017).

As shown in Figure 5D, a one-phase exponential release was investigated in 25 wt% copolymer loaded by ICA at the concentration of 200, 2,000 and 20,000 μM for 24 h, and the release rate of in the three concentrations of ICA reached to 22.63%, 9.02% and 8.58% respectively. Furthermore, sustained release of ICA lasted for 3 weeks and reached to a plateau stage in 200 μM group. Notably, the release of ICA still did not reached to a plateau stage at 4 weeks in both 2,000 and 20,000 μM groups (Figure 5E). The terminal cumulative released amount of ICA from the copolymer at the ICA concentration of 200, 2000 and 20000 μM after 4 weeks was 95.32%, 78.7%, and 75.42% respectively (Figure 5E).

In addition, the release rate of ICA from the copolymer was delayed with an increase in the copolymer concentration from 15 to 25 wt%. Specifically, the release rate of ICA from the copolymer at the concentration of 15, 20 and 25 wt% reached to 89.85%, 82.2%, and 78.7% respectively (Figure 5F).

Based on the above results and considering that the hydrogels degraded more faster with the increase of ICA concentration, the 2000 μM ICA and 25 wt% PLGA-PEG-PLGA hydrogels were applied in the sustained-release drug delivery system to further observe the efficacy of that in the rats model with SONFH.

### The efficacy of ICA co-loaded PLGA-PEG-PLGA hydrogels delivery system combined with core decompression for SONFH

**FIGURE 6:**
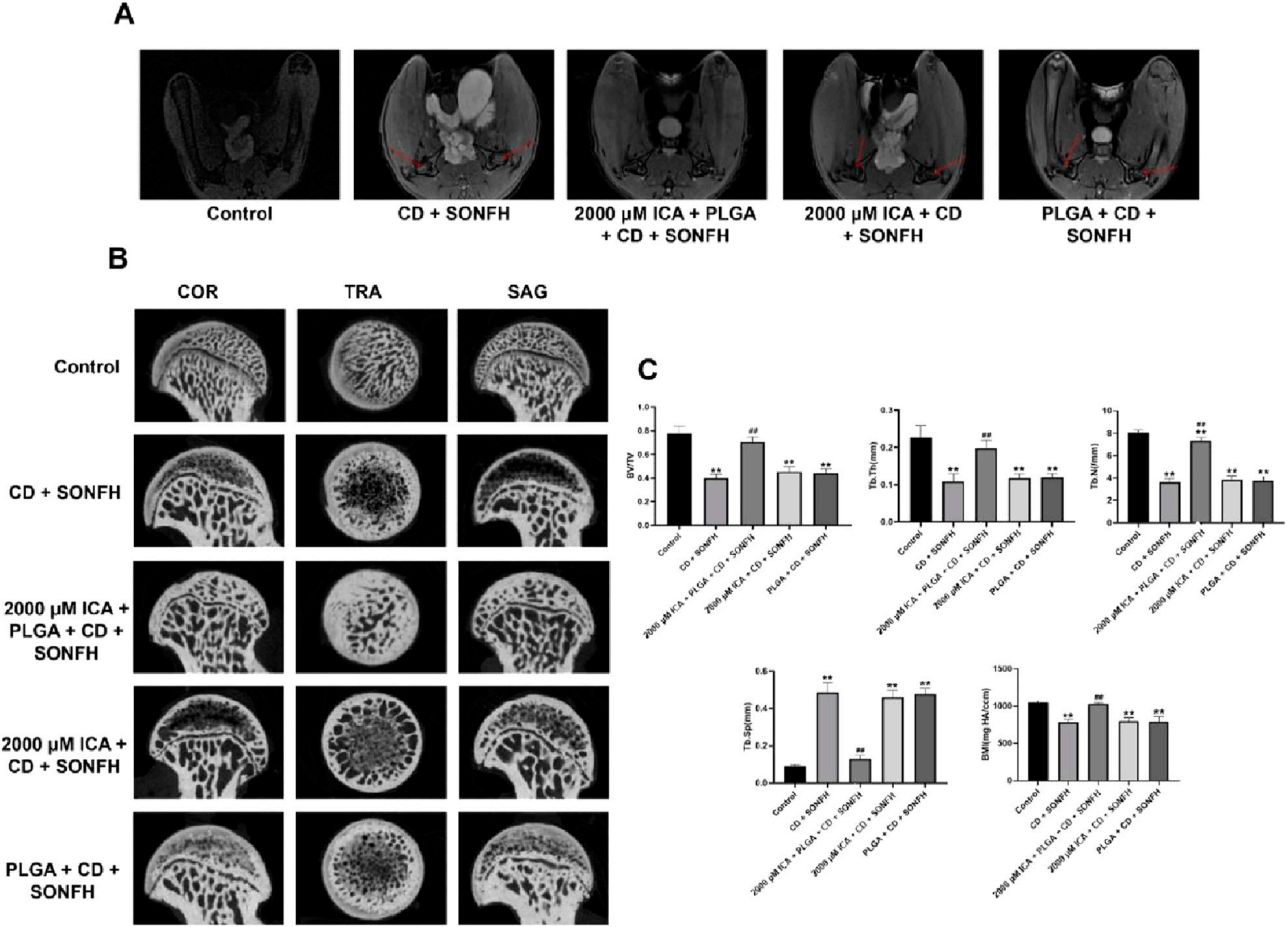
Imaging analysis for the local injection of the sustained-release ICA delivery system co-loaded thermosensitive PLGA-PEG-PLGA hydrogels combined with CD for the treatment of in the rats model with SONFH. **(A)** Representative MRI images. **(B)** Representative micro-CT images. **(C)** Histogram for micro-CT scanning qualitative analysis. Note: all data were expressed as mean ± SD after three replicates. ^**^ *P* < 0.01 compared with the control group, ^##^ *P* < 0.01 compared with the CD + SONFH group, 2,000 μM ICA + CD + SONFH group and PLGA + CD + SONFH group.

The femoral intramedullary injection combined with core decompression was utilized to further reveal the efficacy of sustained-release ICA delivery system co-loaded PLGA-PEG-PLGA hydrogels in the treatment of SONFH. After treatment 12 weeks, a subchondral abnormal high signal showing the bone marrow oedema and necrotic zone in region of the femoral head of rats in all experimental groups was observed in the T2-weighed MRI, indicating that the rats model of early SONFH have been constructed successfully. Surprisingly, subchondral normal signal in the femoral head of rats was found in the 2000 μM ICA + PLGA + CD + SONFH group, however, the remarkable subchondral abnormal high signal still occurred in the CD + SONFH group, PLGA + CD + SONFH group and 2000 μM ICA + CD + SONFH group (Figure 6A). Furthermore, micro-CT scans revealed typical signs of osteonecrosis such as slender or even resorbed bone trabeculaes, bone mass loss and cystic changes in the subchondral region of the rat’s femoral head in the CD + SONFH group, PLGA + CD + SONFH group and 2000 μM ICA + CD + SONFH group. By contrast, these subchondral lesions in the femoral head of rats in the 2000 μM ICA + PLGA + CD + SONFH group were significantly relieved and replaced by the intact and well-distributed trabeculaes (Figure 6B). Further qualitative analyses showed that such microstructural parameters as BV/TV, Tb.Th and Tb.N, were significantly decreased in the rats in the CD + SONFH group, PLGA + CD + SONFH group and 2000 μM ICA + CD + SONFH group except for the increase of Tb. Sp. Whereas, these parameters were reversed in the rats in the 2000 μM ICA + PLGA + CD + SONFH group (Figure 6C). These data showed that the sustained-release ICA delivery system co-loaded PLGA-PEG-PLGA hydrogels combined with core decompression demonstrated to alleviate glucocorticoid-induced bone marrow oedema and osteonecrosis in a rat model.

**FIGURE 7:**
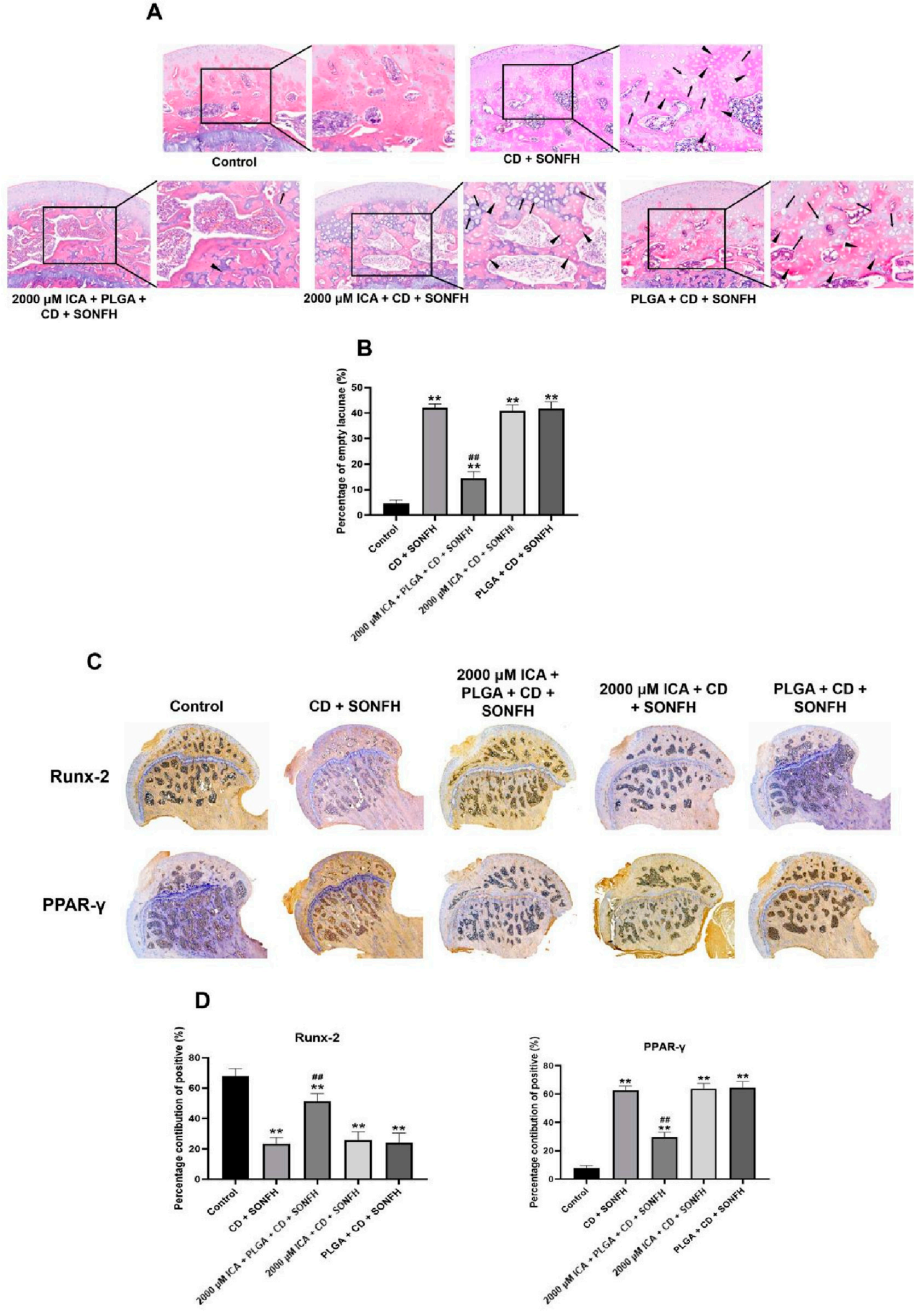
Histology analysis for the local injection of the sustained-release ICA delivery system co-loaded thermosensitive PLGA-PEG-PLGA hydrogels combined with CD for the treatment of in the rats model with SONFH. **(A)** Representative images for HE staining. **(B)** Histogram for percentage of empty lacunae. **(C)** Representative images for IHC analysis. **(D)** Histogram for IHC qualitative analysis. Note: all data were expressed as mean ± SD after three replicates. ^**^ *P* < 0.01 compared with the control group, ^##^ *P* < 0.01 compared with the CD + SONFH group, 2000 μM ICA + CD + SONFH group and PLGA + CD + SONFH group.

Moreover, HE staining analysis showed that the characteristics of osteonecrosis such as the tiny and sparser subchondral trabeculae bone, empty lacunae, as well as the aggregation of adipocytes and increasement of fibrous tissues in the medullary cavity of the femoral head were found in the rats in the CD + SONFH group, PLGA + CD + SONFH group and 2,000 μM ICA + CD + SONFH group. Nevertheless, fewer of the above pathological changes in the subchondral bone of femoral head were observed in the 2000 μM ICA + PLGA + CD + SONFH group (Figure 7A). In addition, a lower rate of empty lacunae of femoral head occurred in the 2000 μM ICA + PLGA + CD + SONFH group compared with the other three groups (Figure 7B).

Meanwhile, IHC staining analysis revealed that higher expression of Runx-2 (an osteogenic marker) and lower expression of PPAR-γ (an adipogenic marker) was observed in the 2000 μM ICA + PLGA + CD + SONFH group, but not in the CD + SONFH group, 2000 μM ICA + CD + SONFH group and PLGA + CD + SONFH group (Figures 7C,D). It was shown that the remarkable promotion of osteogenesis and inhibition of adipogenesis in the rat femoral heads were observed in the 2000 μM ICA + PLGA + CD + SONFH group.

The above findings confirmed that the 2000 μM ICA co-loaded 25 wt% PLGA-PEG-PLGA hydrogels delivery system combined with core decompression has an excellent therapeutic effect on the rats model with SONFH through improving bone crawl replacement and remodelling in the necrotic regions of femoral head.

## Discussion

Both the imbalance of osteogenesis and adipogenesis of BMSCs and the glucocorticoid-induced inhibition of cell proliferation and apoptosis are involved in the pathogenesis of SONFH. Our previous study has conformed the beneficial effects of ICA on regulating the balance between osteogenesis and adipogenesis of BMSCs involving activation of the miR-23a/Wnt/β-catenin signaling pathway axis (Xu et al., 2021a). To our knowledge, few studies have been involved in the anti-apoptotic effect of ICA on the glucocorticoid-induced BMSCs. The present study demonstrated that ICA at the concentration of 10 and 20 μM significantly relieved the Dex-induced apoptosis of BMSCs. However, this anti-apoptotic effect of ICA was weakened prominently at the concentration of 40 μM, which was related to the cytotoxicity of high-dose ICA. A previous report has exhibited the proliferation-inhibition and apoptosis-induction of BMSCs triggered by 40 μM ICA (Wu et al., 2015).

Although the definitive mechanism of Dex-induced apoptosis in BMSCs remains unclear, it appears to be associated with the inhibition of the Akt signalling pathway (Pan et al., 2019; Tao et al., 2017). As a primary mediator of PI3K/Akt signal pathway, Akt are involved in the cell apoptosis, proliferation and migration by activating some signaling cascade (Cantley, 2002; Li et al., 2011; Gu et al., 2013). It is well known that the Akt/Bad/Bcl-2 signaling pathway is taken part to the process of cell apoptosis. Specifically, starting from the phosphorylation of Akt, it causes the inactivation of Bad, and then increases the expression of Bcl-2 protein. This leads to the decrease of caspase-3 activity, and eventually to the inhibition of cell apoptosis (Llambi et al., 2016; Zhu et al., 2014). Our data showed that the phosphorylation of Akt and Bad was promoted, the expression of Bcl-2 was increased, and the activity of caspase-3 was declined in the process of ICA against Dex-induced apoptosis of BMSCs. Interestingly, this anti-apoptotic effect of ICA resembled that of SC79, but could be partly antagonized by MK-2206, indicating that ICA prevented BMSCs from Dex-induced apoptosis through promoting the phosphorylation of Akt/Bad/Bcl-2 signaling pathway.

It was worth noting that ICA has a poor bioavailability after oral administration (Xu et al., 2017), leading to the limited efficacy of traditional administration in the skeletal muscle disorders especially in the SONFH. With the development of tissue engineering technology, the sustained-release drug delivery system co-loaded biodegradable and non-toxic biomaterial provides a promising prospect for the local application of ICA in the treatment of SONFH. PLGA-PEG-PLGA hydrogel as a thermosensitive gelforming copolymer is capable of transforming from solution to gel depending on the phase transition temperature, importantly, exhibits the excellent biodegradability and nontoxicity and is often used as the drug sustained-release carrier (Chan et al., 2019; Ma et al., 2014; Wang et al., 2017; Gao et al., 2011; Gao et al., 2010). In this study, we constructed the sustained-release ICA delivery system co-loaded thermosensitive PLGA-PEG-PLGA hydrogels with a phase transition temperature of 37 °C and evaluated the sol-gel transition and *in vitro* degradation, as well as the sustained release of ICA from this drug delivery system. Our data showed that the degradation of copolymer was related to the ICA concentration *in vitro*, which represented as the faster degradation with the increase of ICA concentration. At the 42nd day, approximately 70% of the 25 wt% copolymers were degraded loading with 2,000 μM ICA. In addition, the sustained-release effect of this delivery system *in vitro* was influenced by the drug or gel concentration. Specifically, the release rate of ICA from the copolymer was negatively correlated with the drug and gel concentration. The terminal cumulative released amount of ICA from the 25 wt% copolymer loading with 2000 μM ICA was 78.7% after 4 weeks. Furthermore, 25 wt% copolymer loading with 2,000 μM ICA also exhibited the sol-gel transition behavior in response to temperature, behaving as a limpid liquid at 4 °C, but formed gel when the environmental temperature reached to 37 °C. Taken together, the sustained-release ICA delivery system co-loaded thermosensitive PLGA-PEG-PLGA hydrogels was constructed using 2000 μM ICA and 25 wt% hydrogels, and utilized in the following experiments *in vivo*.

Core decompression (CD) serves as a traditional surgery for the early SONFH by reducing the pressure in necrotic area of the femoral head directly to provide a biological environment for the repair of necrotic bone. Unfortunately, the clinical efficacy of this technique is still uncertain due to the limited bone regeneration in the decompression area. As result of this, the clinical application of CD is often necessary to implant some fillers in the decompression area, such as autogenous bone, tantalum rod and various biological scaffolds (Xu et al., 2020b). In this study, intramedullary injection of the sustained-release ICA delivery system co-loaded thermosensitive PLGA-PEG-PLGA hydrogels from the distal femur was performed after CD, and systematically evaluated the validity of this surgery in the rats model with SONFH. Surprisingly, based on the MRI, micro-CT scanning and pathological analysis, we found that the local injection of the sustained-release ICA delivery system co-loaded thermosensitive PLGA-PEG-PLGA hydrogels combined with CD could significantly relieve the bone marrow edema, augment the trabeculae bone, reduce the empty lacunae, and decrease the accumulation of adipocyte while increasing the expression of osteogenic marker and inhibiting the expression of adipogenic marker in the femoral head, and eventually promoted the bone repair and remodelling in necrotic areas of the femoral head in the rats model with SONFH.

As a water-insoluble drug, ICA is difficult to absorb in the gastrointestinal tract through traditional oral administration (Xu et al., 2017). The most common way to improve water-insoluble drugs bioavailability is to increase the residence time of drugs in local lesions by using various carriers which are viscous and degradable over a longer time. Due to the adhesive characteristics and hydrophobic-hydrophilic grouping, the thermosensitive PLGA-PEG-PLGA hydrogels used in this study could lead to the slow release of ICA and prolong the retention of ICA in the necrotic area of femoral head by embedding ICA in the gel.

## Conclusion

In summary, this is the first time that ICA has been confirmed to rescue BMSCs from Dex-induced apoptosis through promoting the phosphorylation of Akt/Bad/Bcl-2 signaling pathway. Moreover, local injection of the sustained-release ICA delivery system co-loaded thermosensitive PLGA-PEG-PLGA hydrogels combined with CD could significantly relieve the glucocorticoid-induced early osteonecrosis in the rats model with SONFH by increasing the residence time of ICA in the necrotic area of femoral head to maximize the anti-apoptotic, pro-osteogenic and anti-adipogenic effects of ICA.

## Data Availability

The original contributions presented in the study are included in the article/supplementary material, further inquiries can be directed to the corresponding authors.

## Abbreviations

BMD, bone mineral density; BMSCs, bone mesenchymal stem cells; CD, core decompression; Dex, dexamethasone; EDTA, ethylenediamine tetraacetic acid; FBS, fetal bovine serum; FITC, fluorescein isothiocyanate; GAPDH, glyceraldehyde 3-phosphate dehydrogenase; HE, hematoxylin and eosin; HPLC, high performance liquid chromatography; ICA, icariin; IHC, immunohistochemistry; PE, Phycoerythrin; PEG, polyethylene glycol; PLGA, poly-(_D,L_-lactic acid-co-glycolic acid); PPAR-γ, peroxisome proliferator-activated receptor-γ; PVDF, polyvinylidene difluoride; qRT-PCR, quantitative reverse transcription polymerase chain reaction; Runx-2, runt-related transcription factor-2; SBF, simulated body fluid; SONFH, steroid-induced osteonecrosis of the femoral head; THA, total hip arthroplasty; TM, thrombomodulin; VEGF, vascular endothelial growth factor.
